# Effects of Internal Iliac Artery Ligation on Stress and Urge Incontinence: A Retrospective Evaluation

**DOI:** 10.7759/cureus.63465

**Published:** 2024-06-29

**Authors:** Emrullah Akay, Alime Dilayda Uzun Gül, Enes Burak Mutlu, Ayşe Ceren Nalbant, Kübra Irmak, Fırat Ersan

**Affiliations:** 1 Obstetrics and Gynecology, Başakşehir Çam and Sakura City Hospital, Istanbul, TUR; 2 Obstetrics and Gynecology, Başakşehir Çam and Sakura City Hospital, istanbul, TUR; 3 Obstetrics and Gynecology, Istanbul Istinye University, Istanbul, TUR; 4 Perinatology, Bagcilar Training and Research Hospital, Istanbul, TUR

**Keywords:** stress urinary incontinence, quid test, urge urinary incontinence, internal iliac artery ligation, massive postpartum hemorrhage

## Abstract

Introduction: Does bilateral internal iliac artery ligation (BIIAL), a fundamental intervention in the treatment of postpartum hemorrhage, increase the risk of urinary incontinence (UI)? This study aims to shed light on the effects of BIIAL on bladder perfusion and urinary system integrity, thereby elucidating urinary function disorders following pelvic surgery.

Methods: Demographic and medical data were collected from a total of 192 female patients, with and without the application of BIIAL. Urinary incontinence conditions were assessed using the Questionnaire for Urinary Incontinence Diagnosis (QUID) test. The data collection process was conducted according to ethical standards, and the results were analyzed to determine the types of incontinence.

Results: In the group that underwent BIIAL, the number of pregnancies and births was statistically higher compared to the control group. A significant effect of BIIAL was seen in cases of urge urinary incontinence (UUI), while no meaningful impact was detected on stress urinary incontinence (SUI). After the BIIAL procedure, an increase in the rate of urinary leakage was seen in certain cases.

Conclusion: Bilateral internal iliac artery ligation has proven to be a safe and effective intervention in the management of postpartum hemorrhage. The findings suggest a potential impact of BIIAL on UUI but not on SUI. Comprehensive and long-term prospective studies are needed to further investigate the effects of BIIAL on pelvic blood flow and bladder functions.

## Introduction

Urinary incontinence (UI), with its etiological diversity, is a common health issue affecting many women. It can have negative effects on physical, psychological, and social well-being and may cause significant lifestyle restrictions in some cases [1]. Population-based epidemiological studies have examined the distribution of UI across genders, revealing that it is more prevalent in women than in men [2]. Research considering etiological factors as well as sociodemographic variables shows that approximately 10% of the adult female population experiences symptoms of UI in various forms. This rate can vary with factors such as age, number of births, menopausal status, and history of pelvic surgery, making it a significant public health issue in terms of its impact on women’s health [3].

Stress urinary incontinence (SUI) occurs when involuntary urine leakage happens during moments of increased intra-abdominal pressure, such as coughing, sneezing, or lifting heavy objects. This is often due to the weakness of the pelvic floor muscles and their loss of supportive function. Urge urinary incontinence (UUI) arises from an overactive bladder. In this case, the individual often feels a sudden and uncontrollable need to urinate. This need can be so strong that it leads to an inability to reach the toilet in time, resulting in involuntary urine leakage [4]. The diagnosis of UI begins with various diagnostic tests and assessments; these help to determine the underlying causes of urine leakage and find the most effective treatment strategies.

The Questionnaire for Urinary Incontinence Diagnosis (QUID) test is a short and standardized questionnaire designed to assess symptoms of urine leakage and is considered a reliable method for distinguishing between stress and urge types of urine leakage [5]. This six-question survey is a dependable method for differentiating between stress and urge incontinence and for evaluating the response to treatment [6]. Particularly in resource-limited settings, it stands out as an effective tool for assessing UI [7]. The QUID test objectively measures the severity and frequency of urine leakage symptoms, thus playing a critical role in evaluating the effectiveness of treatment plans and deciding improvement strategies [8].

In conclusion, the QUID test is a valuable tool for assessing symptoms of urine leakage and is widely used in clinical studies. It is a significant instrument for accurately finding types of urine leakage and developing individualized treatment plans. Treatment methods vary depending on the type and severity of UI and include lifestyle changes, exercises, medication therapy, and surgical interventions.

Parity and hysterectomy can increase the risk of UI by weakening the pelvic floor muscles and tissues. As the number of births increases, so does the risk of SUI and UUI. After hysterectomy, disruptions in the supportive structures of the pelvic floor and hormonal changes can particularly increase the risk of moderate to severe UUI. These findings highlight the importance of considering factors such as parity and hysterectomy in the development of prevention and treatment strategies for UI, emphasizing their significance for women’s health [9]. Other variables such as ethnicity, gender, age, obesity, and mode of delivery also play a role in the development of UI. With advancing age, the risk of urine leakage generally increases. Obesity is a significant factor that increases the risk of urine leakage by creating extra pressure on the pelvic floor muscles [10].

Surgical procedures included in treatment protocols, such as hysterectomy and bilateral internal iliac artery ligation (BIIAL), may not be the direct cause of UI but can contribute to its development.

Postpartum hemorrhage is a serious health issue for mothers worldwide and is one of the leading causes of maternal morbidity and mortality. In national and international guidelines, postpartum hemorrhage is typically defined as a blood loss of more than 500 ml in vaginal delivery and 1,000 ml in cesarean delivery. Although the incidence of this condition varies between 1% and 3%, effective management can save mothers’ lives [11].

In cases where medical treatments for postpartum hemorrhage are insufficient, surgical interventions such as ligation of the utero-ovarian and uterine arteries may be applied [12]. However, when hemostasis cannot be achieved, BIIAL can reduce pelvic blood flow, reducing the need for an emergency hysterectomy. Bilateral internal iliac artery ligation is considered a lifesaving method in the control of obstetric and gynecological hemorrhages, and the preservation of uterine and ovarian perfusion, continuation of menstrual cycles, and maintenance of distal arterial flow after this procedure are considered promising findings for future fertility [13].

Bilateral internal iliac artery ligation has been evaluated as an effective and safe method in the treatment of severe postpartum hemorrhage and ongoing bleeding after hysterectomy. This procedure is important because it has a high success rate in achieving hemostasis and reduces the risk of serious complications [14].

This retrospective study examines in depth the effects of BIIAL on bladder blood flow and urinary incontinence in patients with postpartum hemorrhage. The data obtained through the QUID questionnaire allow a broader assessment of urinary dysfunction after pelvic surgery and reveal the role of bladder perfusion and urinary tract integrity in this process. The findings provide important insights that will inform choices of surgical intervention in the management of postpartum hemorrhage and potentially improve clinical practice and patient care standards in this area. Furthermore, this study contributes to the understanding of urinary dysfunction following pelvic surgery, paving the way for the development of new treatment strategies in the field of women's health. 

## Materials and methods

Study design

This study included patients who underwent BIIAL for postpartum hemorrhage between April 1, 2020, and April 20, 2023, and a control group consisting of female patients aged between 15 to 49 years who were treated for postpartum hemorrhage but did not undergo BIIAL in the same period. The data from both groups were retrospectively analyzed.

Study population

The study population consists of a total of 192 patients, including 96 patients with a history of postpartum hemorrhage who underwent BIIAL and 96 patients with a history of postpartum hemorrhage who did not undergo the procedure.

Group comparison

The study compares two groups: the group that underwent BIIAL and the control group. The control group consists of patients who received treatment for postpartum hemorrhage but did not undergo BIIAL. The prevalence of urinary incontinence was assessed based on retrospective data analysis.

Exclusion criteria

Exclusion criteria include individuals who underwent BIIAL for reasons other than postpartum hemorrhage, those who had unilateral ligation, those previously diagnosed with UI, individuals with a BMI over 35 kg/m^2^, those who experienced bladder or ureter injury complications during the operation, individuals who had cesarean delivery due to placenta accreta spectrum or placenta previa, and those who had cesarean hysterectomy.

Follow-up and evaluation

Patients were contacted by phone six months after childbirth or presented to our clinic. Their complaints of urine leakage were inquired about, and the QUID test was administered.

Data collection method

The data collection process was conducted through hospital records and face-to-face interviews, adhering to ethical standards and ensuring patient confidentiality.

The QUID test

The QUID is a test used to evaluate the condition and frequency of urinary incontinence. Participants are asked about their urinary leakage in certain situations, and they are asked to score each situation from 0 (never) to five (always). The test results are divided into two main categories: the stress score, which consists of the sum of questions one, two, and three; and the urgency score, which is the sum of questions four, five, and six. These two scores are used to evaluate different types of urinary incontinence, such as stress and urgency. The sum of both scores provides a general assessment of the individual’s urinary incontinence condition (Table 1).

**Table 1:** The QUID test question: Do you leak urine (even small drops), wet yourself, your pads, or your underwear? Sum responses to items one, two, and three for the stress score; sum responses to items four, five, and six for the urge score. QUID: Questionnaire for Urinary Incontinence Diagnosis

Situation	Never score:0	Rarely score:1	Sometimes score:2	Often score:3	Most of the time score:4	All the time score:5
1. When coughing or sneezing?						
2. When you bend down or lift something up						
3. When you walk quickly, jog, or exercise?						
4. While you are undressing to use the toilet?						
5. Do you get such a strong and uncomfortable need to urinate that you leak urine (even small drops) or wet yourself before reaching the toilet?						
6. Do you have to rush to the bathroom because you get a sudden, strong need to urinate?						

Statistical analysis

Descriptive Statistics

Descriptive statistics for the data included mean, standard deviation, median, minimum, maximum, frequency, and percentage values. The suitability of the variables for a normal distribution was assessed using the Kolmogorov-Smirnov and Shapiro-Wilk tests.

Comparison Between Groups

The BIIAL-applied group consisted of patients who underwent BIIAL and the control group consisted of patients who received treatment for postpartum hemorrhage but did not undergo BIIAL.

Analysis of Independent Data

The comparison between the two groups for quantitative data was made using the Mann-Whitney U test; for qualitative data, the Chi-square test was used.

Analysis Software

Analyses were performed using IBM SPSS Statistics software for Windows, version 28.0 (IBM Corp., Armonk, NY). Depending on the suitability of the data set for normal distribution, appropriate tests were chosen.

Ethics committee approval

Our study received ethical approval following a thorough review and assessment by the ethics committee of Başakşehir Çam and Sakura City Hospital, Istanbul, Türkiye. During the approval process, the purpose, method, potential risks, and participants’ rights of the research were meticulously examined. The ethics committee evaluated the study’s compliance with scientific and ethical standards and approved on August 17, 2023, with the number E-96317027-514.10-222450126 (Subject: KAEK/12.05.2023.212). This approval shows that the study will be conducted with respect for human rights and according to scientific ethical rules. Moreover, it says that ethical principles such as voluntary participation of the participants, informed consent, and the protection of their privacy are prioritized throughout all stages of the study and are governed by the provisions of the Helsinki Declaration.

## Results

Our study reveals a homogeneous distribution in terms of the demographic characteristics of the participants. The average age was found to be 32 years with a standard deviation of ±5.7, showing little variance in ages among the participants and suggesting similarity within the group. The average number of pregnancies (gravida) was 3.5 with a standard deviation of ±1.7, and the average number of births (parity) was 2.9 with a standard deviation of ±1.3. These values show that the participants were also close to each other in terms of the number of pregnancies and births (Table 2).

**Table 2:** Urinary incontinence frequency survey results This table presents the demographic and clinical characteristics of the study population, including key statistics such as age, number of pregnancies, and number of births. Min-max indicates the observed value range. The median is the central number in a data set. The mean±ss shows the average value plus/minus the standard deviation, reflecting the data spread. n-% denotes the count and percentage in the group. Abortion (-/+) signifies if abortions occurred or not. Organ necrosis at ‘0%’ means no observed cases. The total QUID score assesses urinary incontinence severity. The type of incontinence identifies incontinence as none, stress, urge, or mixed. QUID: Questionnaire for Urinary Incontinence Diagnosis

Feature	Min	Max	Median	Average	SD	n	%
Age	18	45	32	32	5.7	-	-
Gravida	1	10	3	3.5	1.7	-	-
Parity	1	6	3	2.9	1.3	-	-
Number of living children	1	6	3	2.9	1.3	-	-
Number of abortions	1	4	1	1.6	0.9	-	-
No abortion	-	-	-	-	-	114	59.40%
Abortion	-	-	-	-	-	78	40.60%
Organ necrosis	-	-	-	-	-	0	0%
Total QUID score	0	20	0	1.4	3.2	-	-
Type of incontinence: none	-	-	-	-	-	149	77.60%
Type of incontinence: stress	-	-	-	-	-	25	13.00%
Type of incontinence: urge	-	-	-	-	-	15	7.80%
Type of incontinence: mixed	-	-	-	-	-	3	1.60%

No cases of organ necrosis were seen in our study; this reflects the effectiveness of the treatment protocols applied, the careful exclusion criteria in participant selection, and the prompt and correct execution of interventions (Table 2).

The total QUID score reflects the frequency and severity of urine leakage among participants. The low average score (1.4 ± 3.2) shows that most participants experienced mild UI symptoms or none. Data on the type and frequency of incontinence show that most participants (77.6%) did not experience any type of incontinence. However, the rates of those experiencing SUI (13.0%) and UUI (7.8%) are important for assessing the prevalence of these types and potential risk factors (Table 3).

**Table 3:** Urinary incontinence frequency survey results This table presents the frequency of urinary incontinence in situations such as coughing or sneezing, bending or lifting a heavy object, walking fast, running, or exercising, undressing to use the toilet, and feeling the need to urinate before reaching the toilet, according to survey results. For each situation, options such as ‘never,’ ‘rarely,’ ‘sometimes,’ ‘often,’ and ‘most of the time’ were indicated, along with the number of participants and percentage rates for each option. n%: This represents the number of individuals in the given category (n) as a percentage of the total number of participants. The “-” symbol indicates that there were no instances or data available for a specific category.

Frequency of urinary incontinence	Never (n%)	Rarely (n%)	Sometimes (n%)	Often (n%)	Most of the time (n%)	All the time (n%)
When coughing or sneezing?	164 (85.4%)	12 (6.3%)	12 (6.3%)	2 (1.0%)	2 (1.0%)	-
When bending down or lifting something up?	167 (87.0%)	12 (6.3%)	11 (5.7%)	1 (0.5%)	-	1 (0.5%)
When walking quickly, jogging, or exercising?	169 (88.0%)	11 (5.7%)	7 (3.6%)	5 (2.6%)	-	-
While undressing to use the toilet?	175 (91.1%)	6 (3.1%)	4 (2.1%)	3 (1.6%)	2 (1.0%)	2 (1.0%)
When you get such a strong and uncomfortable need to urinate that you leak urine (even small drops) or wet yourself before reaching the toilet?	176 (91.7%)	5 (2.6%)	4 (2.1%)	2 (1.0%)	3 (1.6%)	2 (1.0%)
When coughing or sneezing? (Repeat)	177 (92.2%)	3 (1.6%)	3 (1.6%)	5 (2.6%)	1 (0.5%)	3 (1.6%)

No significant difference was found between the control and BIIAL groups in terms of age, number of abortions, and rate of abortion (p > 0.05). However, the BIIAL group showed significantly higher numbers in gravidity, parity, and living children (p<0.05). There was no difference between the two groups about the total QUID score or type of incontinence (p > 0.05). These results suggest that the BIIAL procedure may influence certain demographic characteristics but does not have a significant impact on clinical outcomes (Table 4).

**Table 4:** Comparison of demographic data, total QUID score, and type of incontinence This table compares demographic and incontinence data between the two groups, with notable differences highlighted in the number of births and stress incontinence. The mean is the average of all the data points. The standard deviation (SD) gauges data variability around the mean. Percentage (%) shows the ratio of a category within a group. The median is the central value in an ordered data set. The p-value assesses the likelihood of the null hypothesis being correct, with values below 0.05 typically indicating significance. The chi-square test (X²) evaluates the relationship between two categorical variables. A dash (-) denotes missing or inapplicable data.

Feature	Control group mean	Control group SD	Control group %	Control group median	Internal iliac artery ligations mean	Internal iliac artery ligations SD	Internal iliac artery ligations %	Internal iliac artery ligations Median	p-value
Age	31.4	5.7	-	32	32.6	5.7	-	33	0.196
Gravida	3.2	1.6	-	3	3.9	1.8	-	4	0.006
Parity	2.6	1.3	-	2	3.2	1.2	-	3	0
Abortion (-)	-	-	62.50%	-	-	-	56.30%	-	0.378 (X²)
Abortion (+)	-	-	37.50%	-	-	-	43.80%	-	-
Number of abortions	1.5	0.8	-	1	1.6	1	-	1	0.831
Number of living children	2.6	1.3	-	2	3.2	1.2	-	3	0.001
Total QUID score	1.7	3.8	-	0	1	2.3	-	0	0.81
Type of incontinence: none	-	-	78.10%	-	-	-	77.10%	-	0.863 (X²)
Type of incontinence: stress	-	-	8.30%	-	-	-	17.70%	-	0.863 (X²)
Type of Incontinence: urge	-	-	10.40%	-	-	-	5.20%	-	0.863 (X²)
Type of incontinence: mixed	-	-	3.10%	-	-	-	0.00%	-	0.863 (X²)

According to the data in Table 5, there is no significant difference between the control and BIIAL groups in terms of the rates of urine leakage during coughing, sneezing, bending, lifting heavy objects, walking fast, running, or exercising (p > 0.05). However, the rates of urine leakage while dressing, when feeling a strong urge to urinate, or when experiencing a sudden urge to urinate are significantly higher in the BIIAL group compared to the control group (p < 0.05). These results show that the BIIAL procedure may increase the risk of urine leakage in certain situations. Nevertheless, it appears to have no significant effect on the overall frequency of urine leakage (Table 5).

**Table 5:** Comparison of the QUID test between cases with and without hypogastric artery ligation In the table, n refers to the number of occurrences or individuals within a particular category. The % symbol represents the percentage of the total group that falls into the respective category. The p-value is a statistical measure that indicates the probability of the observed data occurring under the null hypothesis; a lower p-value suggests that the observed data are unlikely under the null hypothesis, often leading to its rejection. The X² symbol denotes the chi-square test, which is used to determine if there is a significant difference between the expected frequencies and the observed frequencies in one or more categories. A dash (-) is used to indicate that a particular data point is not applicable or not provided. QUID: Questionnaire for Urinary Incontinence Diagnosis

Situation	Frequency of urinary incontinence	Control group		Internal iliac artery ligations		p-value	
		n	%	n	%		
When coughing or sneezing?	Never	85	88.50%	79	82.30%	0.22	^X²^
	Rarely	5	5.20%	7	7.30%		
	Sometimes	5	5.20%	7	7.30%		
	Often	0	0.00%	2	2.10%		
	Most of the time	1	1.00%	1	1.00%		
When you bend down or lift something up?	Never	87	90.6%	80	83.3%	0.133	^X²^
	Rarely	4	4.20%	8	8.30%		
	Sometimes	4	4.20%	7	7.30%		
	Often	0	0.00%	1	1.00%		
	All of the time	1	1.00%	0	0.00%		
When you walk quickly, jog or exercise?	Never	86	89.60%	83	86.50%	0.505	^X²^
	Rarely	4	4.20%	7	7.30%		
	Sometimes	3	3.10%	4	4.20%		
	Often	3	3.10%	2	2.10%		
While you are undressing in order to use the toilet?	Never	83	86.5%	92	95.8%	0.022	^X²^
	Rarely	3	3.10%	3	3.10%		
	Sometimes	4	4.20%	0	0.00%		
	Often	2	2.10%	1	1.00%		
	Most of the time	2	2.10%	0	0.00%		
	All of the time	2	2.10%	0	0.00%		
Do you get such a strong and uncomfortable need to urinate that you leak urine (even small drops) or wet yourself before reaching the toilet? ?	Never	83	86.50%	93	96.90%	0.009	^X²^
	Rarely	3	3.10%	2	2.10%		
	Sometimes	4	4.20%	0	0.00%		
	Often	2	2.10%	0	0.00%		
	Most of the time	2	2.10%	1	1.00%		
	All of the time	2	2.10%	0	0.00%		
Do you have to rush to the bathroom because you get a sudden, strong need to urinate?	Never	84	87.50%	93	96.90%	0.016	^X²^
	Rarely	1	1.00%	2	2.10%		
	Sometimes	3	3.10%	0	0.00%		
	Often	5	5.20%	0	0.00%		
	Most of the time	1	1.00%	0	0.00%		
	All of the time	2	2.10%	1	1.00%		

## Discussion

Bilateral internal iliac artery ligation is an essential surgical procedure employed to manage pelvic hemorrhages. This intervention aims to diminish pelvic blood flow by ligating the internal iliac artery, which is critically important due to its potential impact on the vascularization of pelvic organs. In postpartum hemorrhage management, BIIAL is considered a potentially life-saving measure in severe bleeding cases. The procedure is effective in treating and preventing postpartum hemorrhages, particularly reducing the need for hysterectomy in atonic postpartum hemorrhages and aiding surgical repair in traumatic cases [15].

There are studies emphasizing the necessity of a comprehensive understanding of pelvic anatomy and advocating for its inclusion as a core part of medical curricula. These studies assert that BIIAL has a minimal effect on fertility while arguing for its crucial role in obstetrics and gynecology training programs [16]. However, the association of BIIAL with complications such as necrosis of the leg, gluteal muscle, bladder, and rectum, particularly in settings with limited surgical training, has heightened concerns about this technique [17]. Despite adequate collateral blood flow, BIIAL can rarely lead to complete bladder gangrene [18]. Research by Auerbach et al. has elucidated that selective transcatheter arterial embolization of internal iliac artery branches can be safely implemented without causing gluteal necrosis in patients with pelvic trauma [19]. Another study revealed that non-selective internal iliac artery angioembolization could reduce pelvic venous flow in a porcine model but did not completely disrupt it due to the abundant collateral circulation between the external and internal iliac vascular systems [20]. It has been discovered that internal iliac artery ligation (IIAL) prior to myomectomy leads to a decrease in uterine artery resistance and blood flow velocity [21], which could result in reduced tissue oxygenation and potential tissue damage. Over the long term, this reduction in blood flow could affect the functions of reproductive organs, potentially leading to issues such as infertility. Recent studies have shown that IIAL and concurrent hysterectomy can be life-saving interventions in peripartum hemorrhage scenarios. However, these procedures can temporarily reduce serum anti-Müllerian hormone (AMH) levels and ovarian volume in the short term, adversely affecting ovarian reserve [22]. Our study’s findings prove that no serious complications, such as necrosis in vital organs like the gluteal muscles, legs, bladder, ovaries, and uterus, were seen following the BIIAL procedure due to the interruption of blood flow. These results suggest that the BIIAL procedure can be considered a safe therapeutic option in the management of pelvic hemorrhage and has the potential to mitigate concerns about potential risks associated with its implementation. Moreover, these findings support that BIIAL can effectively control blood flow to pelvic organs while preserving their functional integrity, thus making it a preferred intervention method in cases of pelvic hemorrhage (Table 2).

In our study, no statistically significant difference was found between the control group and the BIIAL group in terms of age, number of miscarriages, and abortion rates (p > 0.05). However, in patients with a history of postpartum hemorrhage, the numbers of gravidity and parity were statistically significantly higher in the BIIAL group compared to the control group (p<0.05). The average number of pregnancies in the BIIAL group was 3.9±1.8, while in the control group, this number was 3.2±1.6. These findings suggest that patients who underwent BIIAL had a higher number of pregnancies and births, which could be associated with an increased risk of postpartum hemorrhage. A study by Kaya et al. in the literature reported no statistically significant difference in gravidity and parity between women who underwent internal iliac artery ligation and those who did not [23]. 

Our study presents findings that differ from those in the current literature. It indicates the need for a more detailed examination of the potential role demographic and clinical parameters play in the effects of BIIAL on cases of postpartum hemorrhage. Our results suggest that a higher number of gravidities and parities may increase the risk of postpartum hemorrhage, which in turn could influence the frequency of BIIAL application (Table 4).

The internal iliac artery is the main artery of pelvic blood flow, and the superior vesical artery and internal pudendal artery, which supply blood to the upper and lower parts of the bladder, originate from this artery. Bilateral internal iliac artery ligation could have direct effects on the arteries feeding the bladder. This could affect the elasticity and functionality of the bladder wall. Low blood flow can lead to degeneration of the bladder wall and, therefore, reduce urine retention ability. Internal iliac artery embolization is an effective and minimally invasive method used to stop bladder hemorrhages. Clinical studies where this procedure was applied have seen no long-term complications [24]. A study by Saito et al. on rats proved that BIIAL significantly reduced bladder blood flow and detrusor muscle function, leading to smooth muscle degeneration and, therefore, a loss of bladder wall elasticity and functionality [25]. Chronic bladder ischemia can lead to functional disorders progressing from detrusor overactivity to underactivity over time, associated with changing expression of muscarinic receptors, neural structural damage, and loss of nerve fibers [26]. Other rat studies have revealed that chronic bladder ischemia causes detrusor muscle overactivity [27]. Clinical and experimental studies show that vascular risk factors and bladder ischemia are associated with the severity of lower urinary system symptoms in elderly patients, and this condition can lead to smooth muscle instability and detrusor overactivity in non-obstructive bladders through the activation of cellular stress and survival pathways [28]. Ischemia of the bladder detrusor muscle can trigger lower urinary system symptoms such as stress incontinence and urge incontinence.

No statistically significant relationship was found between patients who underwent BIIAL and the questions related to SUI in the QUID test (p > 0.05), suggesting that BIIAL does not create the expected effect on SUI. On the other hand, in the evaluation of questions related to UUI, a statistically significant difference was found in patients who underwent ligation compared to the control group (p<0.05), showing a potential effect of BIIAL on UUI (Tables 3-5).

The reduction in blood flow following the BIIAL procedure can slow tissue regeneration and healing, potentially prolonging the post-surgical recovery process. Chronic ischemia can affect the long-term health of pelvic organs and contribute to pelvic floor dysfunction. Studies have shown that urinary incontinence is common during pregnancy and the postpartum period, with a prevalence of incontinence seen in about one-fifth of women within the first year after childbirth [29-30]. Our study found that six months after childbirth, the rates of SUI were 17.7% and UUI were 5.2%, which is consistent with the current literature. When the incontinence rates were compared between the BIIAL group and the control group, there was no statistically significant difference, and it was concluded that BIIAL did not have a significant effect on UI (Table 5).

This study on BIIAL and its potential impact on UI postpartum presents several methodological constraints. The limited sample size of 192 may restrict the detection of subtle effects. The short follow-up period may not fully capture long-term outcomes, highlighting the need for extended observation in future research. Reliance on subjective QUID test responses introduces a risk of bias. The study’s generalizability is limited due to its specific demographic and geographic context. Surgical variability and the potential influence of different techniques on outcomes must be considered. Data collection challenges, particularly reliance on patient recall, can affect accuracy. Careful analysis is required to isolate the direct effects of BIIAL from confounding factors, underscoring the need for meticulous interpretation of results. Future studies should aim for larger sample sizes and longer follow-ups to build on these findings.

## Conclusions

The collective evidence from our investigation underscores the role of BIIAL as a practical and secure approach in the management of postpartum hemorrhage without exerting substantial influence on demographic and clinical variables such as age, miscarriage frequency, and hysterectomy occurrence. Crucially, the comparative analysis reveals that BIIAL’s implementation does not escalate the prevalence of SUI or UUI, thereby dispelling concerns over its potential adverse effects on urinary functions.
